# Supplementary material for: Using photovoice to facilitate the report of emotions in an interview setting: An experimental study
Source: PLoS One. 2025 May 6;20(5):e0322055. doi: 10.1371/journal.pone.0322055 (PMC12054863; doi:10.1371/journal.pone.0322055)
Supplement: S1 Appendix — (PDF) [file pone.0322055.s001.pdf]

S1 File. Interview guide.

Hello, thank you for taking the time for this interview. Can you hear me clearly?
I would like to remind you that the interview is being recorded. Do you have any questions about this?
Good, then I would like to begin.

- PVG: I would like you to look at the first picture you sent me.
- WG: I would like to read to you the worry that you sent me.
- CG: I am now going to ask you some questions about worries regarding the future. We can discuss up to five worries here. Please think of your first worry.

I will then ask you some questions about that worry. If you have any questions, feel free to interrupt at at any time.

1. Could you describe the future worry to me in more detail?
2. What does it mean for your life?
3. How do you feel emotionally when you think about it?
   → For example, do you feel nervous, tense, anxious, sad, or angry?
4. Does the future worry trigger anything in your body?
   → For example, do you have palpitations, sweat, or notice pressure in your chest, stomach ache, headache, or the like?
5. How do you deal with the worry? [Behavioral level]
6. What would it need to be for it to be better?

Do you have anything else you would like to say about this worry?

- PVG: Then I would ask you to look at the next picture.
- WG: Then I would now read you the next thought.
- CG: Can you think of any other future worries?

[going through questions 1-6 again.]

We have come to the end of the interview.
Is there anything else from your side now that you would like to get across or comment on?
Thank you again for your participation and your candor. I will now send you a link where you have some questions to answer. This will take about 10 minutes. Answer as spontaneously as possible, there is no right or wrong. Do you have any questions?
Otherwise I thank you and wish you a nice day.
