# Supplementary material for: Using photovoice to facilitate the report of emotions in an interview setting: An experimental study
Source: PLoS One. 2025 May 6;20(5):e0322055. doi: 10.1371/journal.pone.0322055 (PMC12054863; doi:10.1371/journal.pone.0322055)
Supplement: S2 Appendix — (PDF) [file pone.0322055.s002.pdf]

S2 File. Questionnaires.

Perceived difficulty identifying emotions (TAS-DIE)

N°	Item EN	Item GE	Response option
1	I was often confused about what emotion I was feeling.	Mir war oft unklar, was ich gerade fühlte.	5-point Likert scale:<br>Strongly disagree<br>Moderately disagree<br>Neither disagree nor agree<br>Moderately agree<br>Strongly agree
2	I had physical sensations that I didn't understand.	Ich hatte körperliche Empfindungen, die ich selbst nicht verstand.	
3	When I was upset, I didn't know if I was sad, frightened, or angry.	Als ich aufgeregt war, wusste ich nicht, ob ich traurig, ängstlich oder wütend war.	
4	I was often puzzled by sensations in my body.	Ich war oft verwirrt über meine körperlichen Empfindungen.	
5	I had emotions that I couldn't quite identify.	Ich hatte Gefühle, die ich nicht richtig verstehen konnte.	
6	I didn't know what was going on inside me.	Ich wusste nicht, was in mir vorging.	
7	I often didn't know when I was angry.	Ich erkannte oft nicht, wann ich schlecht drauf war.	

Perceived difficulty describing emotions (TAS-DDE)

N°	Item EN	Item GE	Response option
1	It was difficult for me to find the right words for my emotions and thoughts.	Es war schwierig für mich, die richtigen Worte für meine Gefühle und Gedanken zu finden.	5-point Likert scale:<br>Strongly disagree<br>Moderately disagree<br>Neither disagree nor agree<br>Moderately agree<br>Strongly agree
2	I found it easy to describe my emotions and thoughts.	Es fiel mir leicht, meine Gefühle und Gedanken zu beschreiben.	
3	I found it difficult to describe how I felt about people.	Ich fand es schwierig zu beschreiben, wie ich anderen gegenüber fühlte.	

Perceived fear of self-disclosure (STA-R)

N°	Item EN	Item GE	Response option
1	I was embarrassed to tell my interviewer about some of my thoughts and emotions.	Es war mir peinlich, meiner Gesprächspartnerin von manchen Gedanken und Gefühlen zu erzählen.	5-point Likert scale:<br>Strongly disagree<br>Moderately disagree<br>Neither disagree nor agree<br>Moderately agree<br>Strongly agree
2	I found it difficult to talk openly about my thoughts and emotions with my interviewer.	Es fiel mir schwer, mit meiner Gesprächspartnerin offen über meine Gedanken und Gefühle zu sprechen.	
3	I didn't talk about certain emotions because I was afraid of what the interviewer would think about me.	Ich sprach nicht über bestimmte Gefühle, weil ich Angst davor hatte, was meine	

		Gesprächspartnerin über mich denkt.	
4	During the interview, I held back my emotions.	Während des Interviews hielt ich meine Gefühle zurück.	
5	During the interview, I didn't feel like getting involved.	Während des Interviews hatte ich keine Lust mich einzubringen.	

Interviewer's demeanor (INT-DEM)

N°	Item EN	Item GE	
1	friendly	freundlich	7-point Likert scale:<br>not at all<br>to a very great extent
2	similar to me	gleichgesinnt	
3	standoffish (distant and cold in manner)	abweisend (distanziert und kalt)	
4	easy to talk to	einfach mit ins Gespräch zu kommen	
5	unfamiliar	unvertraut	
6	approachable	aufgeschlossen	
7	hard to get along with	schwierig mit auszukommen	
8	trustworthy	vertrauenswürdig	
9	aloof (not friendly or forthcoming)	reserviert (nicht freundlich oder entgegenkommend)	
10	outgoing	aufgeschlossen	
11	unreliable	unzuverlässig	

Quality of relationship (QUAL-REL)

N°	Item EN	Item GE	
1	I was able to bring myself into the interview very well.	Ich konnte mich sehr gut in das Interview einbringen.	5-point Likert scale:<br>Strongly disagree<br>Moderately disagree<br>Neither disagree nor agree<br>Moderately agree<br>Strongly agree
2	During the interview I felt partly superfluous.	Während des Interviews fühlte ich mich teilweise überflüssig.	
3	During the interview, I actively participated in the conversation.	Während des Interviews beteiligte ich mich aktiv an dem Gespräch.	
4	During the interview, I was involved in determining what I wanted to talk about.	Während des Interviews bestimmte ich mit, worüber ich sprechen wollte.	

Perceived interviewer-participant interaction (INTERA)

N°	Item EN	Item GE	
1	well-coordinated	gut abgestimmt, gut koordiniert	7-point Likert scale:<br>not at all<br>to a very great extent
2	boring	langweilig	
3	cooperative	kooperativ (zusammenarbeitend)	
4	harmonious (gets along well with others)	harmonisch	
5	satisfying	zufriedenstellend	
6	comfortably paced	angenehmes Tempo	
7	cold	kalt	

8	awkward	unangenehm	
9	engrossing	spannend	
10	focused	fokussiert	
11	involving	einbeziehend	
12	intense	intensiv	
13	friendly	freundlich	
14	active	lebhaft	
15	positive	positiv	
16	dull	stumpfsinnig	
17	worthwhile	lohnenswert	
18	slow	schleppend, schwerfällig	
